# Supplementary material for: Identification of nanomolar adenosine A2A receptor ligands using reinforcement learning and structure-based drug design
Source: Nat Commun. 2025 Jul 1;16:5485. doi: 10.1038/s41467-025-60629-0 (PMC12216625; doi:10.1038/s41467-025-60629-0)
Supplement: Supplementary file 1 — Supplementary Information [file 41467_2025_60629_MOESM1_ESM.pdf]

Supporting Information for Identification of nanomolar adenosine A_{2A} receptor ligands using reinforcement learning and structure-based drug design

Morgan Thomas[1], Pierre G. Matricon[2], Robert J. Gillespie[2], Maja Napiorkowska[2], Hannah Neale[2], Jonathan S. Mason[2], Jason Brown[2], Kaan Harwood[2], Charlotte Fieldhouse[2], Nigel A. Swain[2], Tian Geng[2], Noel M. O'Boyle[2], Francesca Deflorian[2], Andreas Bender[1,3,4], Chris de Graaf[2,5]

[1] Centre for Molecular Informatics, Department of Chemistry, University of Cambridge, Cambridge, CB2 1EW, UK

[2] Nxera Pharma, Steinmetz Building, Granta Park, Great Abington, Cambridge, CB21 6DG, UK

[3] College of Medicine and Health Sciences, Khalifa University of Science and Technology, Abu Dhabi, United Arab Emirates

[4] STAR-UBB Institute, Babeş-Bolyai University, Cluj-Napoca, Romania

[5] Current affiliation: Structure Therapeutics, 601 Gateway Blvd, San Francisco, CA, 94080, US

Contents

Supplementary Methods 2

Synthesis of Compound 1 3

Synthesis of Compound 2 6

Synthesis of Compound 3 7

Synthesis of Compound 4 9

Synthesis of Compound 5 12

Synthesis of Compound 6 14

Synthesis of Compound 7 17

Synthesis of Compound 8 19

Synthesis of Compound 9 22

Supplementary Figures 26

Supplementary Methods

LC/MS instrumentation and methods

LCMS-05 refers to a Shimadzu Nexera instrument with a photo diode array LC detector and an MS-2020 mass detector.

LCMS-08 refers to an Agilent 1290 RRLC with a diode array LC detector and an Agilent 6120 mass detector.

LCMS-09 refers to an Agilent 1290 RRLC with a diode array LC detector and an Agilent 6120 mass detector.

Method A: Column: X-Bridge C18 50*4.6mm, 3.5µm. Solvent system: (A) 0.1% NH3 in Water. (B)0.1% NH3 in Acetonitrile. Gradient: 95:5 (A:B) at 0.01 min, 10:90 (A:B) at 5.0 min & 5:95 (A:B) at 5.80 min till 7.20min, 95:5 (A:B) at 7.21 min up to 10.0 min. Flow rate: 1.00 mL/min. UV range: 200 – 400 nm. Mass range: 60-1000 amu.

Method B: Column: X-Bridge C18 50*4.6mm, 3.5µm. Solvent system: (A) 5 mM Ammonium bicarbonate in Water. (B) Acetonitrile. Gradient: 95:5 (A:B) at 0.01 min, 15:85(A:B) at 2.8 min & 5:95 (A:B) at 3.5 min till 5.0min, 95:5 (A:B) at 5.01min up to 6.0 min. Flow rate: 1.00 mL/min. UV range: 200 – 400 nm. Mass range: 60-1000 amu.

Synthesis of Compound 1

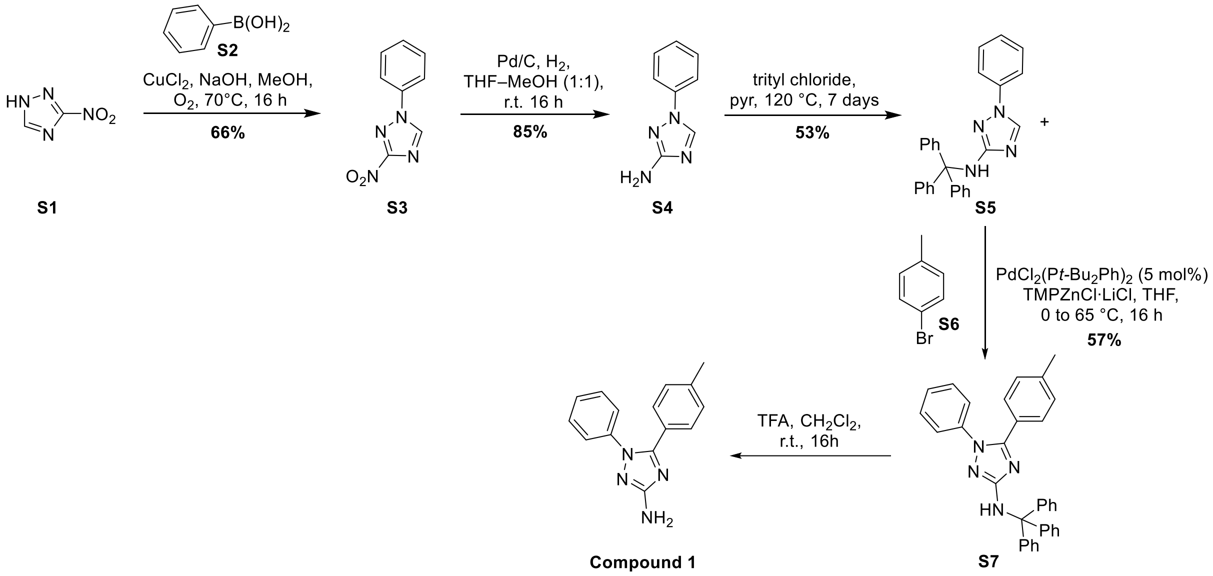

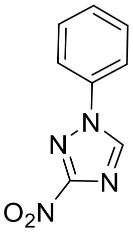

S3

A solution of 3-nitro-1H-1,2,4-triazole (**S1**, 1.00 g, 8.76 mmol, 1.0 equiv), phenylboronic acid (**S2**, 1.74 g, 14.2 mmol, 1.6 equiv), NaOH (0.35 g, 8.76 mmol, 1.0 equiv) and $CuCl_2$ (0.153 g, 1.13 mmol, 12 mol%) in MeOH (20 mL) was aired by O_2 gas for 10 min, the reaction mixture was stirred at 70 °C for 16 h. After completion of reaction, the reaction was filtered through celite bed and washed with ethyl acetate (100 mL) and concentrated under reduced pressure to obtain the crude product (2.67 g). The crude product was purified by flash column chromatography using 230-400 mesh silica gel, eluting ethyl acetate in hexane (0% to 30%) to give 3-nitro-1-phenyl-1H-1,2,4-triazole (**S3**, 1.1 g, 66%) as a yellow solid.

TLC (pet ether:EtOAc, 1:1 v/v): Rf = 0.45; ESI MS (m/z): 191.2 [M+H]+; LC/MS: rt = 2.64 min, 100% peak area at 220 nm (LCMS-08, method A).

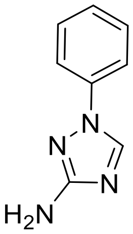

S4

To a solution of 3-nitro-1-phenyl-1H-1,2,4-triazole (**S3**, 1.1 g, 5.78 mmol, 1.0 equiv)) in THF (10 mL) and MeOH (10 mL) was added 10% Pd/C 50% wet (0.2 g) and stirred at rt for 16 h under a H_2 atmosphere. After completion of the reaction, the reaction was filtered through a celite bed and washed with MeOH (100 mL). Concentration under reduced pressure afforded crude 1-phenyl-1H-1,2,4-triazol-3-amine (**S4**, 0.788 g, 85%) as an off-white solid, which was used without any further purification.

TLC (pet ether:EtOAc, 6:4 v/v): Rf = 0.15; ESI MS (m/z): 161.2 [M+H]+; LC/MS: rt = 1.93 min, 100% peak area at 254 nm (LCMS-08, method A).

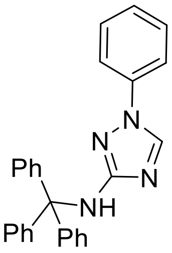

S5

To a solution of 1-phenyl-1H-1,2,4-triazol-3-amine (**S4**, 0.788 g, 4.92 mmol, 1.0 equiv) and trityl chloride (1.5 g, 5.41 mmol, 1.1 equiv) in pyridine (10 mL) was heated at 110 °C for 7 days. After cooling to r.t. the reaction mixture was concentration under reduced pressure to obtain the crude material (2.9 g). The crude product was purified by flash column chromatography using 230-400 mesh silica gel, eluting ethyl acetate in hexane (0% to 27%) to give 1-phenyl-N-trityl-1H-1,2,4-triazol-3-amine (**S5**, 1.06 g, 53%) as a white solid.

TLC (pet ether:EtOAc, 1:1 v/v): Rf = 0.50; ESI MS (m/z): 403.2 [M+H]+; LC/MS: rt = 3.83 min, 90 % peak area at 202 nm (LCMS-08, method A).

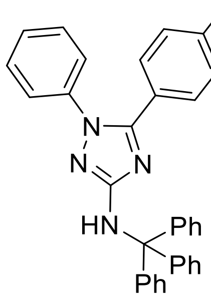

S7

To a 35 mL vial with a stir bar was added 1-phenyl-N-trityl-1H-1,2,4-triazol-3-amine (**S5**, 1.06 g, 2.63 mmol, 1.0 equiv), 1-bromo-4-methylbenzene (**S6**, 0.537 g, 3.16 mmol, 1.2 equiv) and $PdCl_2(Pt\text{-}Bu_2Ph)_2$ (0.163 g, 0.26 mmol, 10 mol%). The vial was sealed with a Teflon-lined cap and THF (3.0 mL) was added. The mixture was cooled in an ice-water bath to 0−5 °C and purged with nitrogen. A solution of TMPZnCl·LiCl in THF (2.23 g, 7.89 mmol, 3.0 equiv) was slowly added, maintaining the temperature at 0 °C. The reaction mixture was then heated to 65 °C for 16 h. After completion of the reaction, the reaction mixture was quenched with cold water (100 mL) and extracted with ethyl acetate (3 x 100 mL). The combined organic extracts were dried over Na_2SO_4 and concentrated under reduced pressure. The crude material was purified by flash column chromatography using 230-400 mesh silica gel, eluting ethyl acetate in hexane (0% to 12%) to give 1-phenyl-5-(p-tolyl)-N-trityl-1H-1,2,4-triazol-3-amine (**S7**, 0.74 g, 57%) as a white solid.

TLC (pet ether:EtOAc, 7:3 v/v): Rf = 0.70; ESI MS (m/z): 493.2 [M+H]+; LC/MS: rt = 4.27 min, 100% peak area at 202 nm (LCMS-08, method A).

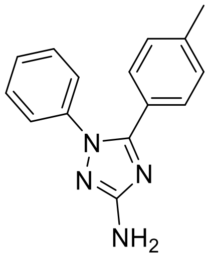

Compound 1

A solution of 1-phenyl-5-(p-tolyl)-N-trityl-1H-1,2,4-triazol-3-amine (**S7**, 0.72 g, 1.46 mmol, 1.0 equiv) in TFA (7.0 mL) was stirred at r.t. for 16h. After completion of reaction, the resulting reaction mixture was quenched with cold sat. aq. $NaHCO_3$ solution (170 mL) and extracted with ethyl acetate (3 x 100 mL). The combined organic extracts were dried over Na_2SO_4 and concentrated under reduced pressure. The crude material was purified by flash column chromatography using 230-400 mesh silica gel, eluting ethyl acetate in hexane (0% to 50%) to give 1-phenyl-5-(p-tolyl)-1H-1,2,4-triazol-3-amine (**Compound 1**, 184 mg, 50%) as an off-white solid.

ESI MS (m/z): 251.2 [M+H]+; 1H NMR (400 MHz, DMSO-d6): δ 7.42 – 7.39 (m, 3H), 7.28 – 7.23 (m, 4H), 7.17 – 7.15 (m, 2H), 5.57 (s, 2H), 2.29 (s, 3H); 13C NMR (101 MHz, DMSO-d6): δ 163.9, 152.3, 139.8, 138.8, 129.7, 129.5, 128.7, 128.4, 125.9, 125.6, 21.3; LC/MS: rt = 2.68 min, 100% peak area at 202 nm (LCMS-08, method A).

Synthesis of Compound 2

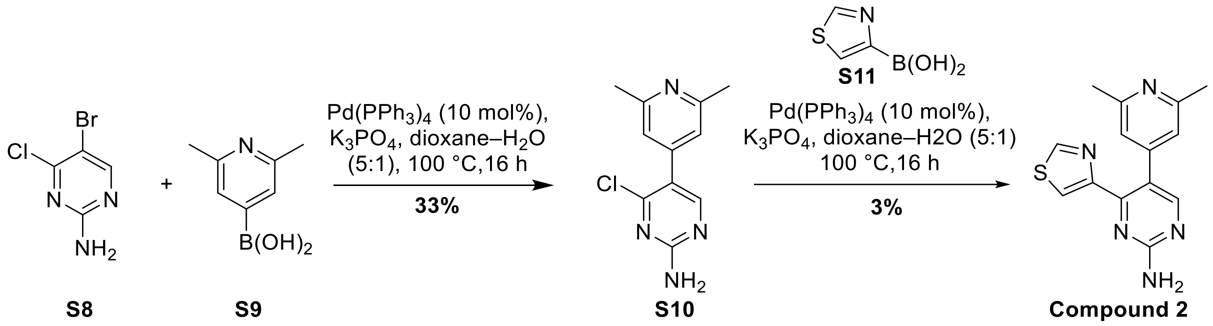

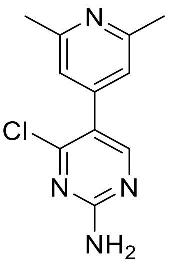

S10

To a stirred solution of 5-bromo-4-chloropyrimidin-2-amine (**S8**, 500 mg, 2.42 mmol, 1.0 equiv) and 2,6-dimethylpyridin-4-yl) boronic acid (**S9**, 0.432 g, 2.84 mmol, 1.17 equiv) in dioxane (5.0 mL) and water (1.0 mL), was added K_3PO_4 (1.0 g, 4.81 mmol, 1.99 equiv). The reaction mixture was degassed with nitrogen for 15 min. To the reaction mixture $Pd(PPh_3)_4$ (0.027 mg, 0.24 mmol, 10 mol%) was added. After further degassing with nitrogen (5 mins), the reaction mixture was heated to 100 °C and stirred for 16 h. After completion, the reaction mixture was diluted with water (20 mL) and extracted with ethyl acetate (2 x 50 mL). The combined organic extracts were dried over anhydrous Na_2SO_4 filtered and concentrated under reduced pressure to give a crude residue. The crude material was purified by flash chromatography using 0%-50% EtOAc in hexane as an eluent to give 4-chloro-5-(2,6-dimethylpyridin-4-yl) pyrimidin-2-amine (**S10**, 210 mg, 33%) as a white solid.

TLC (EtOAc:hexanes, 1:1 v/v): Rf = 0.50; ESI MS (m/z): 235.4 [M+H]+; 1H NMR (400 MHz, DMSO-d6): δ 8.46 (s, 1H), 7.65–7.55 (m, 7H), 7.22 (s, 1H), 7.07 (s, 2H); LC/MS: rt = 2.11 min, 6 % peak area at 254 nm (LCMS-09, method B).

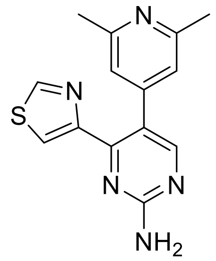

Compound 2

To a stirred solution of 4-chloro-5-(2,6-dimethylpyridin-4-yl) pyrimidin-2-amine (**S10**, 150 mg, 0.64 mmol, 1.0 equiv) and 2,6-dimethylpyridin-4-yl) boronic acid (**S11**, 0.065 g, 0.51 mmol, 0.8 equiv) in dioxane (5.0 mL) and water (1.0 mL) was added K_3PO_4 (0.270 g, 1.28 mmol, 2.0 equiv) at r.t. The reaction mixture was degassed with nitrogen for 15 min. To the reaction mixture $Pd(PPh_3)_4$ (0.05 mg, 0.0064 mmol, 10 mol%) was added. After further degassing with nitrogen for 5 mins, the reaction mixture was stirred at 100 °C for 16 h. After completion, the reaction mixture was diluted with water (20 mL) and extracted with ethyl acetate (2 x 50 mL). The combined organic extracts were dried over anhydrous Na_2SO_4, filtered and concentrated under reduced pressure to give crude product. The crude material was purified by prep-HPLC to give the title compound 5-(2,6-dimethylpyridin-4-yl)-4-(thiazol-4-yl) pyrimidin-2-amine (0.057 mg, 3.14 %) as an off white solid.

Prep HPLC method: The compound was purified on Shimadzu LC-20AP and UV detector. The column used was X-Bridge C8(250*19) mm, 5 µ, Column flow was 12.0 ml/min. Mobile phases were used (A)5mM Ammonium Bicarbonate and 0.1% NH3 in Water and (B) 100% Acetonitrile. The gradient solvent B was 0-22 % over 25 min, 100 % over 31 min then 100-0% over 0 min.

TLC (EtOAc:hexanes, 8:2 v/v): Rf = 0.50; ESI MS (m/z): 284.4 [M+H]+; 1H NMR (400 MHz, DMSO-d6): δ 9.05 (s, 1H), 8.55 (s, 1H), 7.38 (s, 1H), 7.04 (s, 2H), 6.89 (s, 2H), 2.35 (s, 6H); 13C NMR (101 MHz, DMSO-d6): δ 163.3, 163.1, 160.3, 157.4, 154.4, 151.5, 147.2, 119.9, 117.0, 116.4, 24.4; LC/MS: rt = 1.94 min, 100% peak area at 202 nm (LCMS-08, method B)

Synthesis of Compound 3

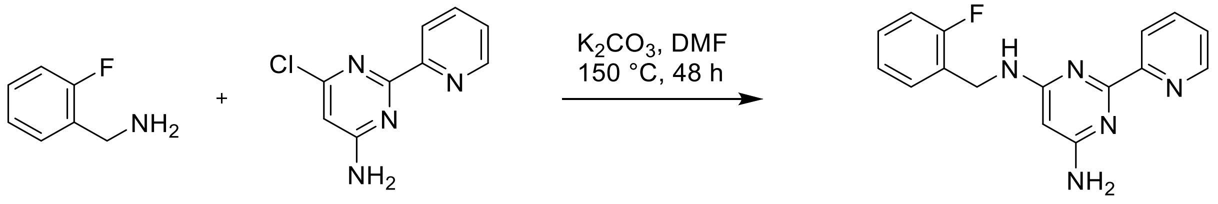

S12 **S13** **Compound 3**

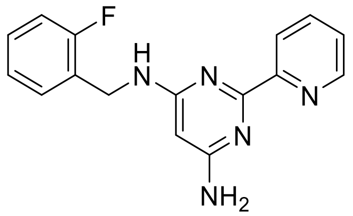

Compound 3

To a stirred solution of (2-fluorophenyl) methanamine (454 mg, 3.63) in DMF (5.0 mL) was added K_2CO_3 (668.9 mg, 4.84 mmol) and KI (602.6 mg, 3.63 mmol) and stirred RM at RT for 30 min. Then after 7-6-chloro-2-(pyridin-2-yl) pyrimidin-4-amine (500 mg, 2.42 mmol) was added and stirred RM at 150 °C for 48h. The completion of the reaction was monitored by TLC, the reaction mixture quenched with water (20 mL) and extracted with EtOAc (2 X 30 ml), the organic layer was dried over Na_2SO_4 and concentrated under high vacuum to afford crude. The crude product was purified by prep HPLC the pure fractions were evaporated under high vacuum to afford pure N4-(2-fluorobenzyl)-2-(pyridin-2-yl) pyrimidine-4,6-diamine (260 mg, 36%) as a white solid.

Prep-HPLC Method: The compound was purified on Shimadzu LC-20AP and UV detector. The column used was UNFIRE C18(250*19) mm, 5µ. Column flow was 15 ml/min. Mobile phase were used (A) 0.1% TFA IN WATER (B) 100% ACN. The gradient solvent B was 5-40% over 27 min, 40-100% over 2min then 100%-0% over 6 min.

13C NMR Method: This was run by VT at 75 °C to collapse doubled signals.

TLC (EtOAc:hexane, 7:3 v/v): Rf = 0.45; ESI MS (m/z): 296.2 [M+H]+; 1H NMR (400 MHz, DMSO-d6): δ 8.80 (d, J = 8.0 Hz , 1H), 8.57 (s, 1H), 8.38 (d, J = 8.0 Hz, 1H), 8.12 (t, J = 8.0 Hz, 1H), 7.73 (t, J = 8.0 Hz, 1H), 7.52 (s, 1H), 7.45 (t, J = 8.0 Hz, 1H), 7.34 – 7.36 (m, 1H), 7.23 – 7.18 (m, 2H), 5.75 (s, 1H), 4.69 – 4.67 (m, 2H); 13C NMR (101 MHz, DMSO-d6): δ 161.9, 160.8 (d, J = 245.1 Hz), 156.6, 154.3, 149.7, 147.8, 138.9, 130.3 (d, J = 4.2 Hz), 129.9 (d, J = 8.1 Hz), 128.2, 125.3 (d, J = 16.1 Hz), 125.0 (d, J = 3.5 Hz), 123.3, 115.8 (d, J = 21.2 Hz), 80.8, 39.1; 19F NMR (376 MHz, DMSO-d6): δ -73.5; LC/MS: rt = 2.36 min, 100 % peak area at 190 nm (LCMS-05, method A).

Synthesis of Compound 4

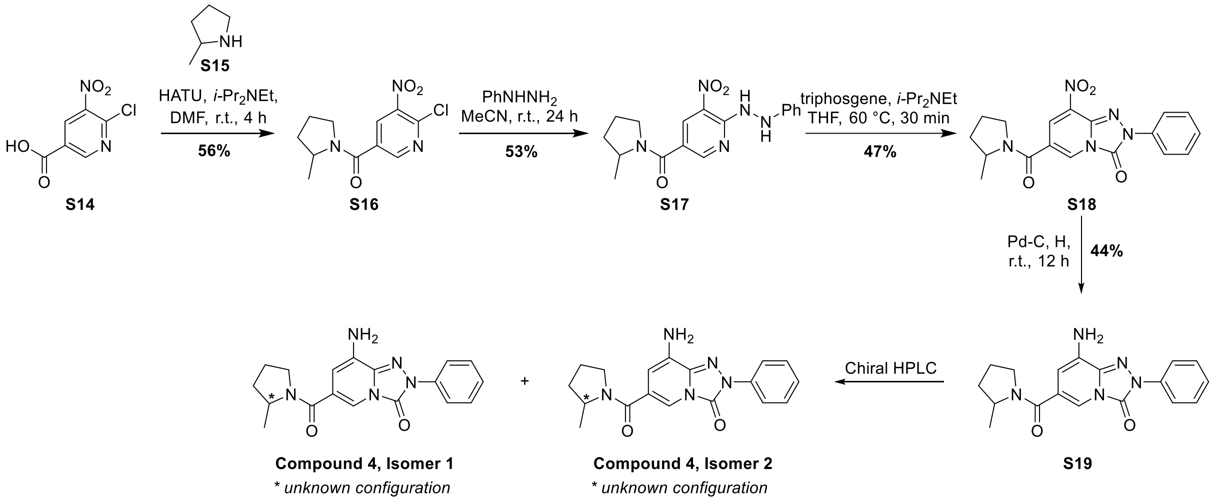

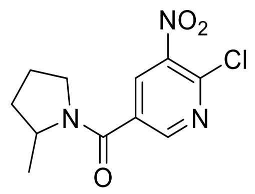

S16

To a stirred solution of 6-chloro-5-nitronicotinic acid (**S14**, 2.00 g, 9.90 mmol, 1.0 equiv) in DMF (20 mL) was added *i*-Pr_2NEt (5.04 mL, 29.70 mmol, 3.0 equiv) and HATU (5.64 g, 14.85 mmol, 1.5 equiv) and the reaction mixture was stirred for 5 min. 2-methyl pyrrolidine (**S15**, 841.5 mg, 9.90 mmol, 1.0 equiv) was then added and the reaction mixture stirred at r.t. for 4 h. The reaction mixture was diluted with H_2O (30 mL), and the aqueous layer was extracted with EtOAc (2 x 50 mL). The combined organic extracts were dried over Na_2SO_4 and concentrated under reduced pressure. The crude material was purified by column chromatography (100-120 mesh Silica gel, 0% to 40% EtOAc in Hexane) to afford (6-chloro-5-nitropyridin-3-yl)(2-methylpyrrolidin-1-yl)methanone (**S16**, 1.50 g, 56%) as off white solid.

TLC (EtOAc:hexane, 1:1 v/v): Rf = 0.40; ESI MS (m/z): 270.2 [M+H]+; LC/MS: rt = 2.78 min, 86 % peak area at 220 nm (LCMS-08, method A).

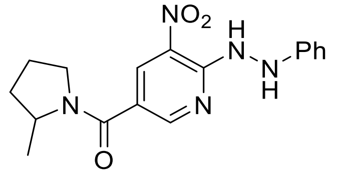

S17

To a stirred solution of (6-chloro-5-nitropyridin-3-yl)(2-methylpyrrolidin-1-yl)methanone (**S16**, 1.50 g, 5.57 mmol, 1.0 equiv) in MeCN (20 mL) was added phenyl hydrazine (602 mg, 5.57 mmol, 1.0 equiv)

and stirred the reaction mixture at r.t. for 24 hr. The reaction mixture was diluted with H_2O (30 mL) and the aqueous layer was extracted with EtOAc (2 x 50 mL). The combined organic extracts were dried over Na_2SO_4 and solvent was removed under vacuum. The crude material was purified by column chromatography (100-120 mesh Silica gel, 0% to 60% EtOAc in Hexane) to afford (2-methylpyrrolidin-1-yl)(5-nitro-6-(2-phenylhydrazineyl)pyridin-3-yl)methanone (**S17**, 1.0 g, 53%) as a yellow solid.

TLC (EtOAc:hexane, 1:1 v/v): Rf = 0.30; ESI MS (m/z): 342.2 [M+H]+; LC/MS: rt = 2.87 min (26 %) and 2.91 min (74 %) peak areas at 240 nm (LCMS-08, method A).

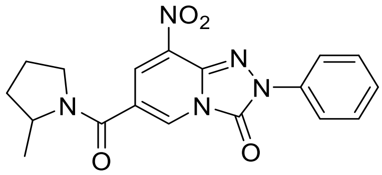

S18

To a stirred solution of (2-methylpyrrolidin-1-yl) (5-nitro-6-(2-phenylhydrazineyl) pyridin-3-yl) methanone (**S17**, 1.00 g, 2.93 mmol, 1.0 equiv) and *i*-Pr_2NEt (0.50 mL, 2.93 mmol, 1.0 equiv) in THF (20 mL) was added triphosgene (867 mg, 2.93 mmol, 1.0 equiv). The reaction mixture was stirred at at 60 °C for 30 min. After this time, the reaction mixture was poured in sat. aq. $NaHCO_3$ solution (15 mL) and diluted with H_2O (20 mL). The aqueous layer was extracted with EtOAc (2 x 30 mL) and the combined organic extracts were dried over Na_2SO_4 and concentrated under reduced pressure. The crude material was purified by column chromatography (100-120 mesh Silica gel, 0% to 60% EtOAc in hexane) to afford 6-(2-methylpyrrolidine-1-carbonyl)-8-nitro-2-phenyl-[1,2,4]triazolo [4,3-a]pyridin-3(2H)-one (**S18**, 0.500 g, 47%) as a yellow solid.

TLC (EtOAc:hexane, 1:1 v/v): Rf = 0.40; ESI MS (m/z): 368.2 [M+H]+; LC/MS: rt = 2.97 min, 100 % peak area at 220 nm (LCMS-08, method A).

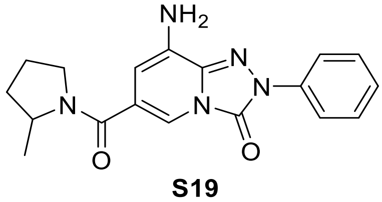

S19

To a stirred solution of 6-(2-methylpyrrolidine-1-carbonyl)-8-nitro-2-phenyl-[1,2,4]triazolo[4,3-a]pyridin-3(2H)-one (**S18**, 0.500 g, 1.36 mmol, 1.0 equiv) in MeOH (20 mL), was added palladium on carbon (10% w/v, 226 mg, 0.136 mmol, 10 mol%). The reaction mixture was stirred at r.t. under a H_2

atmosphere for 16 h. The reaction mixture was passed through celite pad washed with 10% MeOH in CH_2Cl_2. The filtrate was concentrated under reduced pressure and the crude material then purified by column chromatography (100-120 mesh Silica gel, 0% to 60% EtOAc in Hexane) to afford 8-amino-6-(2-methylpyrrolidine-1-carbonyl)-2-phenyl-[1,2,4]triazolo[4,3-a]pyridin-3(2H)-one (**S19**, 0.200 g, 44%) as off white solid.

Two enantiomers were separated by chiral SFC purification using CHIRALPAK AD-H (250*21) mm, 5u column, and LIQUID.CO2 and 0.1% DEA IN IPA: ACN (50:50) as eluent.

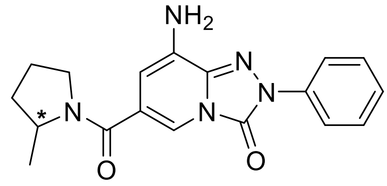

Compound 4, Isomer 1

** unknown configuration*

TLC (EtOAc:hexane, 1:1 v/v): Rf = 0.30; ESI MS (m/z): 338.2 [M+H]+; 1H NMR (400 MHz, DMSO-d6): δ 8.07 (d, J = 7.6 Hz, 2H), 7.53 (t, J = 7.6 Hz, 2H), 7.40 (s, 1H), 7.31 (s, 1H), 6.28 (s, 1H), 6.05 (s, 2H), 4.11–4.09 (m, 1H), 3.51–3.45 (m, 2H), 2.09–2.01 (m, 1H), 1.88–1.82 (m, 1H), 1.74–1.68 (m, 1H), 1.54 (s, 1H), 1.22 (s, 3H); 13C NMR (101 MHz, DMSO-d6): δ 165.8, 147.8, 138.1, 137.5, 134.8, 129.6, 126.4, 123.6, 119.6, 111.2, 102.8, 53.5, 49.9, 32.8, 25.0, 20.0; LC/MS: rt = 2.66 min, 100 % peak area at 225 nm (LCMS-08, method A).

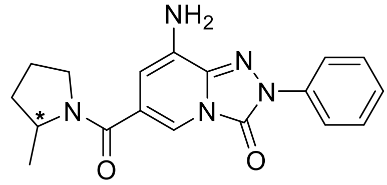

Compound 4, Isomer 2

** unknown configuration*

TLC (EA:Hexane, 1:1 v/v): Rf = 0.30; ESI MS (m/z): 338.2 [M+H]+; 1H NMR (400 MHz, DMSO-d6): δ 8.09 (d, J = 7.6 Hz, 2H), 7.55 (t, J = 7.6 Hz, 2H), 7.43 (s, 1H), 7.34 (d, J = 7.6 Hz, 1H), 6.29 (s, 1H), 6.06 (s, 2H), 4.13-4.12 (m, 1H), 3.53 (s, 2H), 2.11-2.01 (m, 1H), 1.89 (s, 1H), 1.74-1.68 (m, 1H), 1.56-1.55 (m, 1H), 1.25-1.21 (m, 3H); 13C NMR (101 MHz, DMSO-d6): δ 165.8, 147.8, 138.1, 137.5, 134.8, 129.6, 126.4, 123.6, 119.6, 111.1, 102.8, 53.5, 49.9, 32.8, 25.0, 20.0; LC/MS: rt = 2.66 min, 100 % peak area at 225 nm (LCMS-08, method A).

Synthesis of Compound 5

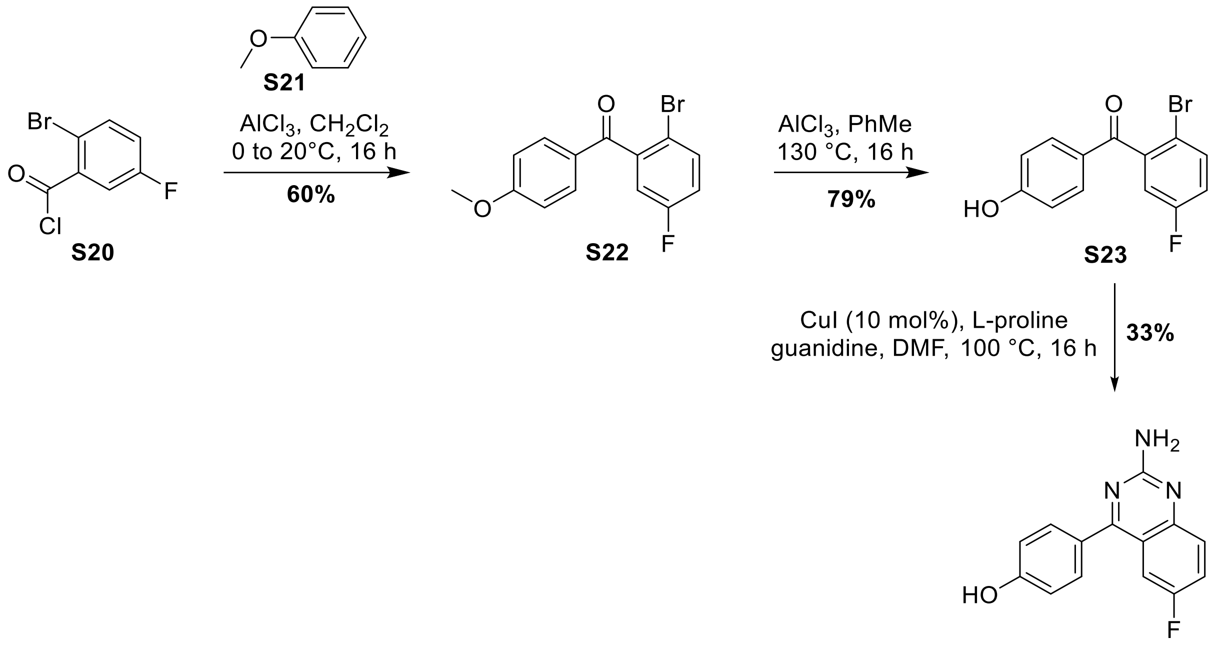

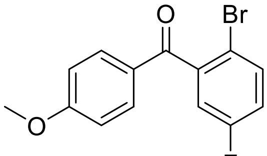

S22

To a stirred solution of 2-bromo-5-fluorobenzoyl chloride (**S20**, 1.00 g, 4.26 mmol, 1.0 equiv) and Anisole (**S21**, 0.546 g, 5.06 mmol, 1.2 equiv) in CH_2Cl_2 (10 mL), was added $AlCl_3$ (0.674 g, 5.06 mmol, 1.2 equiv) portion wise at 0 °C. The reaction mixture was warmed to r.t. and stirred for 16 h. The reaction mixture was quenched with 4N HCl (20 mL) and extracted with CH_2Cl_2 (3 x 60 mL). The combined organic extracts were dried over Na_2SO_4 and concentrated under reduced pressure. The crude material was triturated with petroleum ether to obtain (2-bromo-5-fluorophenyl) (4-methoxyphenyl) methanone (**S22**, 0.800 g, 60%) as a white solid.

TLC (Ethyl acetate:Hexane, 2:8 v/v): Rf = 0.3; ESI MS (m/z): 309.2 [M+H]+; 1H NMR (400 MHz, CDCl3): δ 7.80 (d, J = 8.8 Hz, 2H), 7.60 (m, 1H), 7.08 (d, J = 8.0 Hz, 2H), 6.96 (d, J = 8.4 Hz, 2H), 3.9 (s, 3H); LC/MS: rt = 3.46 min, 94 % peak area at 254 nm (LCMS-09, method A).

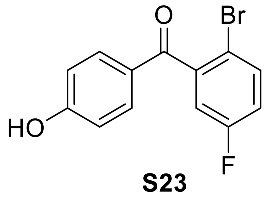

S23

To a stirred solution of (2-bromo-5-fluorophenyl) (4-methoxyphenyl) methanone (**S22**, 0.500 g, 1.69 mmol, 1.0 equiv) in Toluene (5.0 mL), was added $AlCl_3$ (0.539 g, 4.05 mmol, 2.4 equiv) portion wise at 0 °C. The reaction mixture was then stirred at 130 °C for 16 h. The reaction mixture was diluted with water (100 mL) and extracted with ethyl acetate (3 x 60 mL). The combined organic extracts were dried over anhydrous Na_2SO_4 and concentrated under reduced pressure. The crude material was purified by flash chromatography (0 to 70% ethyl acetate in hexane) to give (2-bromo-5-fluorophenyl) (4-hydroxyphenyl) methanone (**S23**, 0.380 g, 79%) as a brown solid.

TLC (EtOAc:Hexane, 4:6 v/v): Rf = 0.20; ESI MS (m/z): [M+H]+; 1H NMR (400 MHz, CDCl3): δ 7.80 (d, J = 8.8 Hz, 2H), 7.60 (m, 1H), 7.08 (d, J = 8.0 Hz, 2H), 6.96 (d, J = 8.4 Hz, 2H), 4.16 (s, 1H); LC/MS: rt = 2.91 min, 100 % peak area at 254 nm (LCMS-08, method A).

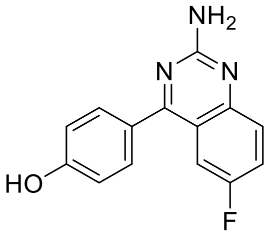

Compound 5

A solution of (2-bromo-5-fluorophenyl) (4-hydroxyphenyl) methanone (**S23**, 0.380 g, 1.29 mmol, 1.0 equiv), guanidine (0.151 g, 2.56 mmol, 1.98 equiv) and caesium carbonate (0.837 g, 2.56 mmol, 1.98 equiv) in DMF (5.0 mL) was degassed with nitrogen for 15 min. CuI (0.122 g, 0.644 mmol, 50 mol%) and L-proline (0.074 g, 0.644 mmol, 50 mol%) were then added. The reaction mixture was stirred at 100 °C for 16 h. After this time, it was diluted with water (40 mL) and extracted with ethyl acetate (3 x 50 mL). The combined organic extracts were washed with cold water (3 x 50 mL) then dried over anhydrous Na_2SO_4 and concentrated under reduced pressure. The crude material was purified by flash chromatography (0% to 100% ethyl acetate in hexane) to give 4-(2-amino-6-fluoroquinazolin-4-yl)phenol (**Compound 5**, 0.110 g, 33% yield) as a brown solid.

TLC (EtOAc:hexane, 4:6 v/v): Rf = 0.20; ESI MS (m/z): 256.2 [M+H]+; 1H NMR (400 MHz, CDCl3): δ 9.90 (s, 1H), 7.80 (d, J = 8.0 Hz, 2H), 7.60 (m, 1H), 7.63 – 7.55 (d, J = 8.2 Hz, 4H), 6.96 (d, J = 8.0 Hz, 2H), 6.82

(s, 2H); 13C NMR (101 MHz, DMSO-d6): δ 168.9 (d, J = 4.9 Hz), 160.6, 159.6, 156.9 (d, J = 239.8 Hz), 150.9, 131.6, 128.3 (d, J = 8.1 Hz), 127.9, 123.7 (d, J = 25.2 Hz), 117.4 (d, J = 8.3 Hz), 115.8, 110.8 (d, J = 22.8 Hz); 19F NMR (376 MHz, DMSO-d6): δ -119.2; LC/MS: rt = 2.35 min, 99 % peak area at 202 nm (LCMS-08, method A).

Synthesis of Compound 6

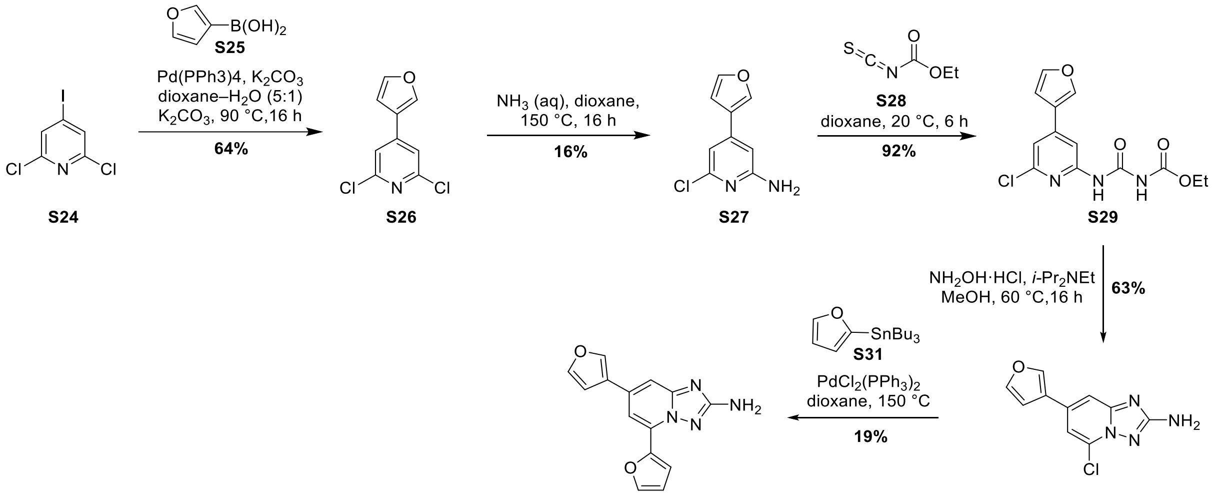

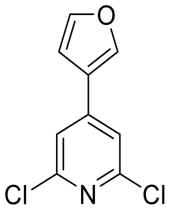

S26

To a stirred solution of 2,6-dichloro-4-iodopyridine (**S24**, 2.00 g, 7.35 mmol, 2.0 equiv) and 2-(furan-3-yl)-4,4,5,5-tetramethyl-1,3,2-dioxaborolane (**S25**, 0.713 g, 3.68 mmol, 1.0 equiv) in 1,4-dioxane (10 mL) and water (2 mL), was added K_2CO_3 (2.0 g, 14.7 mmol, 4.0 equiv) at r.t.. The reaction mixture was degassed with nitrogen for 15 min. To the reaction mixture $Pd(PPh_3)_4$ (0.027 mg, 0.2403 mmol, 6.5 mol%) was added. After the reaction degassed with N_2 for 5 min. The reaction mixture was stirred at 90 °C for 16 h. After completion, the reaction mixture was diluted with water (50 mL) and extracted with EtOAc (2 x 70 mL). The combined organic extracts were dried over anhydrous Na_2SO_4 and concentrated under reduced pressure. The crude material was purified by flash chromatography (0%-20% EtOAc in hexane) as an eluent to give 2,6-dichloro-4-(furan-3-yl) pyridine (**S26**, 1.0 g, 64%) as a colourless gummy liquid.

TLC (EtOAc:Hexanes, 2:8 v/v): Rf = 0.40; ESI MS (m/z): 216.0 [M+H]+; LC/MS: rt = 3.38 min, 55 % peak area at 240 nm (LCMS-08, method B).

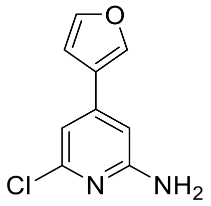

S27

To a stirred solution of 2,6-dichloro-4-(furan-3-yl) pyridine (**S26**, 1.00 g, 9.35 mmol, 1.0 equiv) in Dioxane (10.0 mL), was added 28% aq. ammonia (10 mL) at r.t. The reaction mixture was stirred at 150 °C for 16 h. After completion, the reaction mixture was diluted with water (50 mL) and extracted with EtOAc (2 x 60 mL). The combined organic layer was dried over anhydrous Na_2SO_4 filtered and concentrated under reduced pressure to give a crude residue. The crude material was purified by flash chromatography (0%-20% EtOAc in hexane) to give 6-chloro-4-(furan-3-yl) pyridin-2-amine (**S27**, 0.150 g, 16%) as a white solid.

TLC (EtOAc:Hexanes, 1:1 v/v): Rf = 0.20; ESI MS (m/z): 195.2 [M+H]+; LC/MS: rt = 2.74 min, 91 % peak area at 202 nm (LCMS-08, method B).

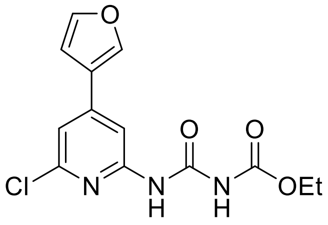

S29

To a stirred solution of 6-chloro-4-(furan-3-yl) pyridin-2-amine (**S27**, 0.340 g, 1.75 mmol, 1.0 equiv) and *O*-ethyl carbonisothiocyanatidate (**S28**, 0.459 g, 3.51 mmol, 2.0 equiv) in dioxane (5.0 mL) at 0 °C. The reaction mixture was stirred at RT for 6 h. The reaction mixture was directly concentrated under reduced pressure to give the crude product 6-chloro-4-(furan-3-yl) pyridin-2-ethyl urea (0.50 g, 92%) as a brown solid. The crude product was used without further purification.

TLC (EtOAc:Hexane, 3:7 v/v): Rf = 0.30; ESI MS (m/z): 328.0 [M+H]+; LC/MS: rt = 3.60 min, 35 % peak area at 220 nm (LCMS-08, method B).

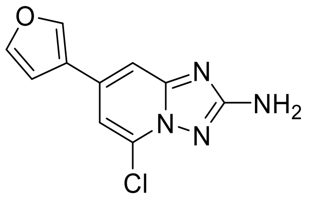

S30

To a stirred solution of 6-chloro-4-(furan-3-yl) pyridin-2-ethyl urea (**S29**, 0.500 g, 1.62 mmol, 1.0 equiv) in methanol (10 mL), was added hydroxylamine hydrochloride (0.223 g, 3.23 mmol, 2.0 equiv) and *i*-Pr_2NEt (0.84 mL, 4.85 mmol, 3.0 equiv) drop wise at 0 °C. The reaction mixture was then stirred at 60 °C for 16 h. The reaction mixture was directly evaporated, diluted with water (20 mL) and extracted with ethyl acetate (3 x 40 mL). The combined organic extracts were filtered over dried anhydrous Na_2SO_4 and concentrated under reduced pressure. The crude material was purified by flash chromatography (0-50% EtOAc in hexane) to give 5-chloro-7-(furan-3-yl)-[1,2,4] triazolo[1,5-a]pyridin-2-amine (**S30**, 0.240 g, 63%) as a colourless gummy liquid.

TLC (EtOAc:Hexane, 2:8 v/v): Rf = 0.30; ESI MS (m/z): 235.2 [M+H]+; 1H NMR (400 MHz, CDCl3): δ 8.47 (d, J = 6.0 Hz, 1H), 7.81 (s, 1H), 7.65 (s, 1H), 7.50 (s, 1H), 7.19 (s, 1H), 6.28 (s, 2H); LC/MS: rt = 2.28 min, 100% peak area at 220 nm (LCMS-08, method B).

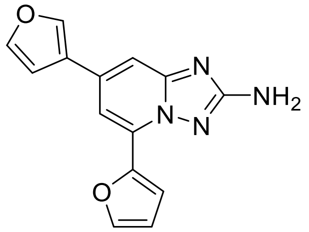

Compound 6

A stirred solution of 5-chloro-7-(furan-3-yl)-[1,2,4] triazolo[1,5-a]pyridin-2-amine (**S30**, 0.220 g, 0.94 mmol, 1.0 equiv) and tributyl(furan-2-yl)stannane (**S31**, 0.671 g, 1.88 mmol, 2.0 equiv) in 1,4-dioxane (10 mL) at r.t. was degassed with nitrogen for 15 min. To the reaction mixture was added Bis (triphenylphosphine)palladium (II) dichloride (0.032 mg, 0.047 mmol, 0.05 equiv). The reaction mixture was stirred at 150 °C for 16 h. After completion, the reaction mixture was diluted with water (20 mL) and extracted with ethyl acetate (2 x 30 mL). The combined organic extracts were dried over anhydrous Na_2SO_4, filtered and concentrated under reduced pressure. The crude material was purified by flash chromatography (0-50% EtOAc in hexane) to give the 5-(furan-2-yl)-7-(furan-3-yl)-[1,2,4] triazolo[1,5-a]pyridin-2-amine (**Compound 6**, 0.013 g, 19%) as a white solid.

TLC (EtOAc:Hexanes, 3:7 v/v): Rf = 0.30; ESI MS (m/z): 267.2 [M+H]+; 1H NMR (400 MHz, DMSO-d6): δ 8.52 (s, 1H), 8.03 (d, J = 3.2 Hz, 1H), 7.89 (d, J = 3.2 Hz, 1H), 7.81 (d, J = 3.6 Hz, 1H), 7.62 (d, J = 2 Hz, 1H), 7.58 (d, J = 1.6 Hz, 1H), 7.21 (d, J= 1.6 Hz, 1H), 6.83 (s, 1H), 6.19 (s, 2H); 13C NMR (101 MHz, DMSO-d6): δ 166.9, 152.1, 145.5, 145.2, 145.2, 141.8, 132.9, 129.2, 124.7, 115.8, 113.1, 109.2, 106.3, 105.0; LC/MS: rt = 2.74 min, 100% peak area at 254 nm (LCMS-08, method B).

Synthesis of Compound 7

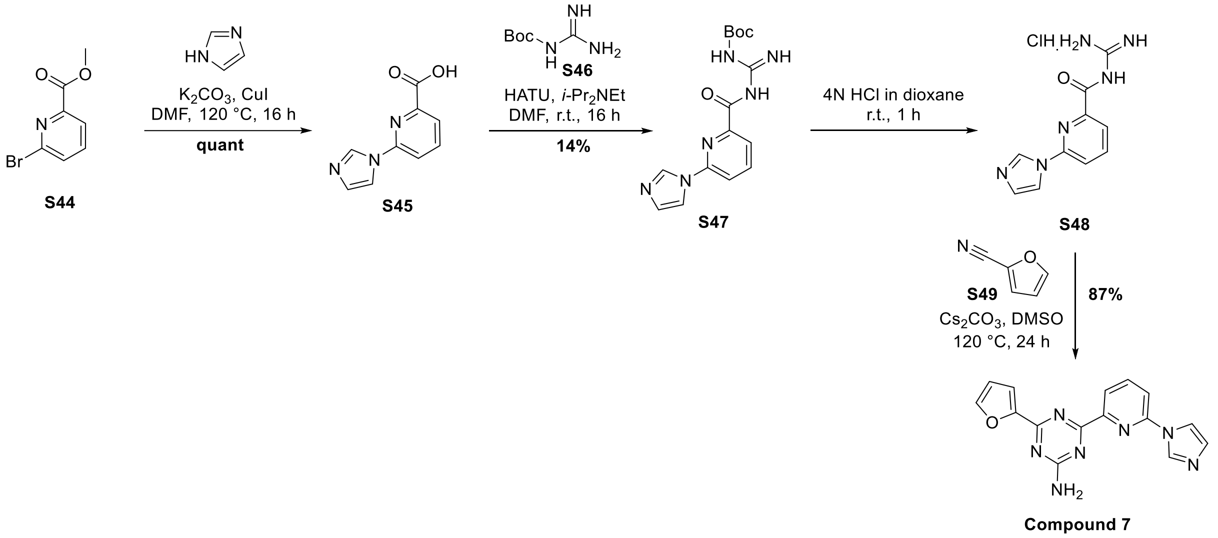

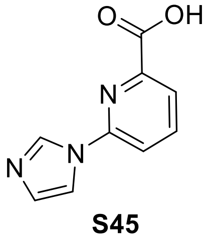

S45

To a degassed solution of methyl 6-bromopicolinate (**S44**, 0.5 g, 2.33 mmol, 1.0 equiv) and 1H-imidazole (0.69 g, 6.97 mmol, 3.0 equiv) in DMF (10 mL) was added K_2CO_3 (0.80 g, 5.81 mmol, 2.5 equiv) and CuI (40 mg, 0.21 mmol, 0.1 equiv). The reaction mixture was heated at 120 °C for 16 h. After completion, the reaction mixture was filtered through celite and washed with 10% MeOH in CH_2Cl_2 (150 mL). The combined filtrates were concentrated under reduced pressure. The crude material was purified by trituration with pentane and diethyl ether (1:1) to give 6-(1H-imidazol-1-yl) picolinic acid (**S45**, 0.75 g, quant) as a pale-yellow solid.

TLC (EtOAc): Rf = 0.15; ESI MS (m/z): 190.4 [M+H]+; 1H NMR (400 MHz, DMSO-d6): δ 12.14 (bs, 1H), 8.49 (s, 1H), 7.93 (s, 1H), 7.86 (d, J = 8.0 Hz, 1H), 7.69 (d, J = 8.0 Hz, 1H), 7.63-7.61 (m, 2H); LC/MS: rt = 0.92 min, 73 % peak area at 220 nm (LCMS-09, method B).

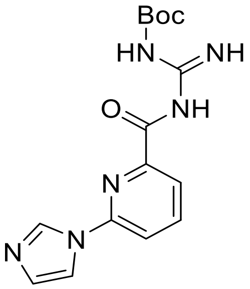

S47

To a stirred solution of 6-(1H-imidazol-1-yl)picolinic acid (**S45**, 0.75 g, 3.97 mmol, 1.0 equiv) in DMF (5.0 mL) was added HATU (2.3 g, 5.95 mmol, 1.5 equiv) at r.t. The reaction mixture was stirred for 30 min at this temperature then cooled to 0 °C. Boc-Guanidine (0.946 g, 5.95 mmol, 1.5 equiv) was then added slowly at 0 °C then followed by the addition of *i*-Pr_2NEt (2.05 mL, 11.90 mmol, 3.0 equiv) at 0 °C and then the resulting reaction mixture was stirred at 25 °C for 16 h. The reaction mixture was then poured into cold H_2O (50 mL) which resulted in a white precipitate which was filtered and dried under reduced pressure to afford N'-Boc-N-carbamimidoyl-6-(1H-imidazol-1-yl) picolinamide (**S47**, 180 mg, 14%), as a light yellow solid.

TLC (EtOAc): Rf = 0.40; ESI MS (m/z): 331.4 [M+H]+; LC/MS: rt = 2.47 min, 91% peak area at 220 nm (LCMS-09, method B).

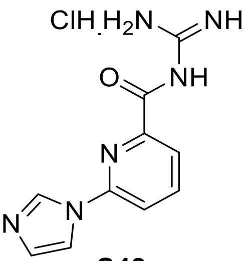

S48

To a solution of N'-boc-N-carbamimidoyl-6-(1H-imidazol-1-yl) picolinamide (**S47**, 0.16 g, 0.48 mmol, 1.0 equiv) in dioxane (2 mL) at 0 °C was added 4M HCl in dioxane (1.5 mL). The reaction mixture was warmed to rt and stirred for 1 h. After this time, the reaction mixture was concentrated under reduced pressure to afford a crude solid. This solid was triturated with pentane (5 mL) to give N-carbamimidoyl-6-(1H-imidazol-1-yl) picolinamide hydrochloride (**S48**, 0.17 g) as a white solid.

TLC (EtOAc): Rf = 0.40; ESI MS (m/z): 231.4 [M+H]+; LC/MS: rt = 1.57 min, 29 % peak area at 210 nm (LCMS-09, method B).

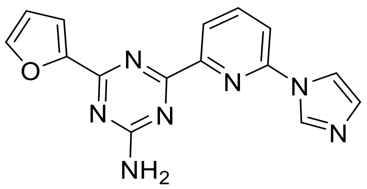

Compound 7

To a stirred solution of furan-2-carbonitrile (**S49**, 30 mg, 0.32 mmol, 1.0 equiv) and N-carbamimidoyl-6-(1H-imidazol-1-yl) picolinamide hydrochloride (**S48**, 129 mg, 0.48 mmol, 1.5 equiv) in DMSO (2 mL) was added Cs_2CO_3 (315 mg, 0.97 mmol, 3.0 equiv). The reaction mixture was then was heated at 120 °C for 24 h The reaction mixture was poured into cold water (15mL) and extracted with 10% MeOH in DCM (10 mL X 3). The organic layer was dried over sodium sulfate, filtered, and concentrated under reduced pressure to give Crude (120 mg). The crude product was purified by normal phase combi-flash column chromatography in silica, at 0% to 10% (MeOH in DCM) to give a pure compound. Which was further re-purified by prep. HPLC to give (30 mg, 87.00%) as a white solid.

PREP HPLC Method: The compound was purified on Shimadzu LC-20AP and UV detector. The column used was X-BRIDGE C8(250*20) mm,5µ. Column flow was 13.0 ml/min. The mobile phase was used (A) 5mM ABC+ 0.1% NH3 IN WATER and (B) 100% Acetonitrile. The gradient solvent B was 0-35% over 25 min, then 35% over 3 min, 100-100% over 2 min, and 0% over 6 min.

TLC (EtOAc): Rf = 0.30; ESI MS (m/z): 306.2 [M+H]+; 1H NMR (400 MHz, DMSO-d6): δ 8.62 (s, 1H), 8.35 (d, J = 8.0 Hz, 1H), 8.21 (t, J = 8.0 Hz, 1H), 8.05-8.00 (m, 3H), 7.85 (s, 2H), 7.51 (d, J = 3.2 Hz, 1H), 7.19 (s, 1H), 6.76-6.75 (m, 1H); 13C NMR (101 MHz, DMSO-d6): δ 170.1, 167.9, 164.3, 153.6, 151.2, 149.1, 147.3, 141.1, 135.7, 130.6, 122.6, 117.3, 116.5, 115.6, 113.1; LC/MS: rt = 2.11 min, 100% peak area at 304 nm (LCMS-08, method B).

Synthesis of Compound 8

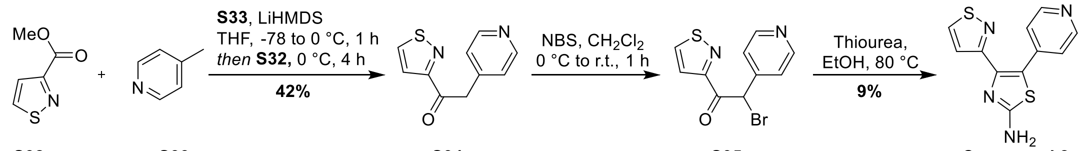

S32 S33 S34 S35 Compound 8

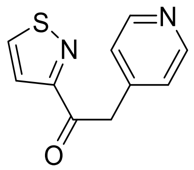

S34

A stirred solution of 4-methylpyridine (**S33**, 0.3 g, 3.22 mmol, 1.0 equiv) was dissolved in anhydrous THF (2 mL) under a nitrogen atmosphere. The reaction mixture was cooled to -78°C and LiHMDS (0.5 M in Toluene, 12.9 mL, 6.45 mmol, 2.0 equiv) was added dropwise while stirring. The reaction mixture was allowed to warm up to 0 °C, stirred for 1 h and then cooled to -78°C. Methyl isothiazole-3-carboxylate (**S32**, 0.69 g, 4.83 mmol, 1.5 equiv) in anhydrous THF (3 mL) was added. The resulting reaction mixture was stirred at 0 °C for 4 h. Upon completion, the reaction mixture was diluted with saturated aq. ammonium chloride solution (50 mL) and EtOAc (30 mL). The aqueous layer was extracted with ethyl acetate (30 mL X 2) and the combined organic extracts were dried over Na_2SO_4, and concentrated under reduced pressure. The crude material was purified by column chromatography (0% to 70% (EtOAc in Hexane) to give 1-(isothiazol-3-yl)-2-(pyridin-4-yl)ethan-1-one (**S34**, 180 mg, 42 %), as a white solid.

TLC (hexane:EtOAc, 7:3 v/v): Rf = 0.20; ESI MS (m/z): 205.3 [M+H]+; 1H NMR (400 MHz, DMSO-d6): δ 9.18 (d, J = 4.0 Hz, 1H), 8.50 (d, J = 8.0 Hz, 2H), 7.84 (d, J = 4.0 Hz, 1H), 7.30 (d, J = 4.0 Hz, 2H), 4.55 (s, 2H); LC/MS: rt = 2.24 min, 100 % peak area at 202 nm (LCMS-08, method A).

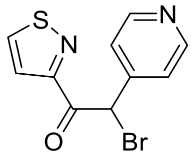

S35

To a stirred solution of 1-(isothiazol-3-yl)-2-(pyridin-4-yl)ethan-1-one (**S34**, 170 mg, 0.83 mmol, 1.0 equiv) in CH_2Cl_2 (1.0 mL) at 0 °C was added NBS (130 mg, 0.83 mmol, 1.0 equiv). The reaction mixture was then warmed to r.t. and stirred for 1 h. Upon completion, the reaction mixture was diluted with water (30 mL) and EtOAc (20 mL). The aqueous layer was extracted with ethyl acetate (20 mL X 2) and the combined organics dried over Na_2SO_4 and concentrated under reduced pressure to give crude 2-bromo-1-(isothiazol-3-yl)-2-(pyridin-4-yl)ethan-1-one (**S35**, 110 mg) as a light yellow solid which was used without any further purification.

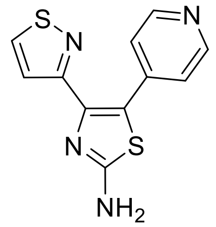

Compound 8

To a stirred solution of 2-bromo-1-(isothiazol-3-yl)-2-(pyridin-4-yl)ethan-1-one (**S35**, 110 mg, 0.39 mmol, 1.0 equiv) in EtOH (2 mL) was added thiourea (45 mg, 0.59 mmol, 1.5 equiv). The reaction mixture was warmed to 80 °C and stirred for 3 h. After cooling to r.t., the reaction mixture was concentrated under reduced pressure and the crude material suspended between water (15mL) and 10% MeOH in CH_2Cl_2 (10 mL). The aqueous layer was extracted with 10% MeOH in CH_2Cl_2 (10 mL X 3) and the combined organic extracts were dried over Na_2SO_4 and concentrated under reduced pressure. The crude material was purified by flash column chromatography (0 to 10% MeOH in CH_2Cl_2) to give crude material (80 mg) which was further re-purified by prep. HPLC to give 4-(isothiazol-3-yl)-5-(pyridin-4-yl)thiazol-2-amine (**Compound 8**, 7.9 mg, 9%) as a white solid.

Prep-HPLC Method: The compound was purified on Shimadzu LC-20AP and UV detector. The column used was X-BRIDGE C8(250*19)mm,5µ. Column flow was 14.0 ml/min. Mobile phase were used (A) 5MM ABC+0.1%NH_3 in water and (B) 100% Acetonitrile. The gradient solvent B was 0-30% over 28 min, 30% over 1min, then 100% over 2 min, 100-0% over 6 min.

TLC (EtOAc): Rf = 0.15; ESI MS (m/z): 261.4 [M+H]+; 1H NMR (400 MHz, DMSO-d6): δ 9.10 (d, J = 4.0 Hz, 1H), 8.47-8.45 (m, 2H), 7.55 (d, J = 4.0 Hz, 1H), 7.50 (s, 2H), 7.29-7.28 (m, 2H); 13C NMR (101 MHz, DMSO-d6): δ 167.8, 163.3, 150.7, 149.6, 142.9, 140.6, 125.4, 123.5, 120.2; LC/MS: rt = 1.94 min, 99 % peak area at 304 nm (LCMS-09, method B).

Synthesis of Compound 9

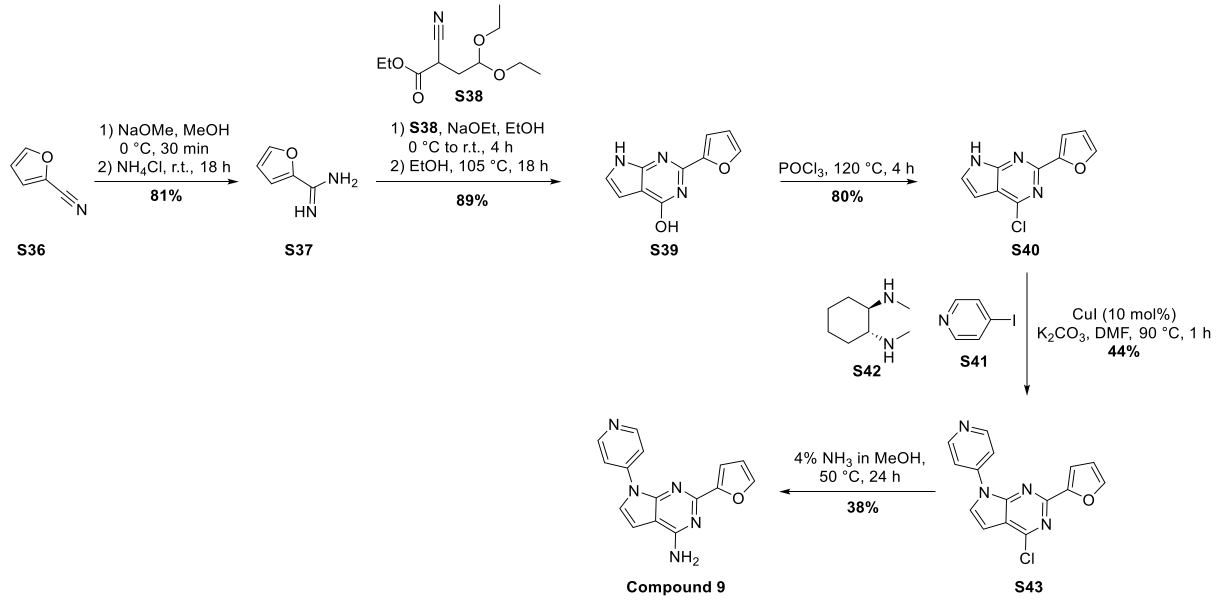

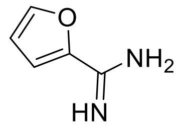

S37

To a stirred solution of furan-2-carbonitrile (**S36**, 5.0 g, 53.7 mmol, 1.0 equiv) in methanol (20 mL) at 0 °C was added sodium methoxide (4.35 g, 80.56 mmol, 1.5 equiv). The reaction mixture was then stirred at 0 °C for 30 mins. Ammonium chloride (8.62 g, 161.2 mmol, 3.0 equiv) was then added and the reaction mixture was stirred at r.t. for 18 h. The mixture was then filtered and the filter bed washed with MeOH. The filtrate was dried over Na_2SO_4 and concentrated under reduced pressure give crude furan-2-carboximidamide (**S37**, 4.8 g, 81%) as a white solid which was used without any further purification.

TLC (MeOH:CH2Cl2, 1:9 v/v): Rf = 0.20; ESI MS (m/z): 111.2 [M+H]+; LC/MS: rt = 0.65-0.77 min, 83% peak area at 254 nm (LCMS-08, method B).

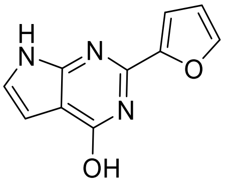

S39

To a stirred solution of furan-2-carboximidamide (**S37**, 1.0 g, 9.08 mmol, 1.0 equiv) and ethyl 2-cyano-4,4-diethoxybutanoate (**S38**, 3.12 g, 13.6 mmol, 1.5 equiv) in ethanol (3.0 mL) at 0 °C was added 21% sodium ethoxide in ethanol (5.80 mL, 18.16 mmol, 2.0 equiv) dropwise. The reaction mixture was

stirred at r.t. for 4 h. Ethanol (5.0 mL) was added and the reaction was heated at 105 °C for 18 h. After cooling to r.t., the reaction mixture was concentrated under reduced pressure. The residue was suspended in water (100 mL) and extracted with ethyl acetate (2x50 mL). The combined organic extracts were washed with brine (50 mL), dried over anhydrous Na_2SO_4 and concentrated under reduced pressure. The crude material was purified by flash chromatography (neutral silica, 0-100% EtOAc in hexanes) to give 2-(furan-2-yl)-7H-pyrrolo[2,3-d] pyrimidin-4-ol (**S39**, 1.6 g, 89%) as an off-white solid.

TLC (MeOH:DCM, 1:9 v/v): Rf = 0.40; ESI MS (m/z): 202.4 [M+H]+; 1H NMR (400 MHz, DMSO-d6): δ 4.60 (t, J = 6.8 Hz, 1H), 3.66 – 3.58 (m, 2H), 3.46 – 3.39 (m, 2H), 2.59 – 2.58 (m, 2H), 1.10 – 1.03 (m, 6H); LC/MS: rt = 1.71 min, 89% peak area at 220 nm (LCMS-09, method B).

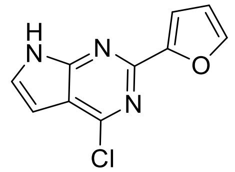

S40

A mixture of 2-(furan-2-yl)-7H-pyrrolo[2,3-d] pyrimidin-4-ol (**S39**, 5.0 g, 15.7 mmol) in $POCl_3$ (3.0 mL) was heated to 120 °C and stirred for 4 h. After cooling to r.t., the reaction mixture was concentrated under reduced pressure. The residue was poured into ice water (50 mL) where a black precipitate formed. The precipitated solids were filtered and washed with water (2 x 20 mL). The crude solids were then purified by flash chromatography (neutral silica, 0-30% EtOAc in hexanes) to give 4-chloro-2-(furan-2-yl)-7H-pyrrolo[2,3-d] pyrimidine (**S40**, 1.3 g, 80%) as a white solid.

TLC (EtOAc:hexanes, 1:1 v/v): Rf = 0.70; ESI MS (m/z): 220.2 [M+H]+; 1H NMR (400 MHz, DMSO-d6): δ 12.57 (bs, 1H), 7.90 (d, J = 2.0 Hz, 1H), 7.78 (d, J = 3.6 Hz, 1H), 7.24 (d, J = 3.2 Hz, 1H), 6.70 (d, J = 3.2 Hz, 1H),6.61 (t, J = 3.6 Hz, 1H); LC/MS: rt = 2.80 min, 98% peak area at 202 nm (LCMS-09, method B).

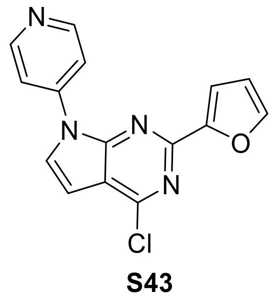

S43

4-chloro-2-(furan-2-yl)-7H-pyrrolo[2,3-d]pyrimidine (**S40**, 500 mg, 2.28 mmol, 1.0 equiv) was dissolved

in DMF (5 mL) followed by the addition of 4-iodopyridine (**S41**, 936 mg, 4.56 mmol, 2.0 equiv), K_2CO_3 (945 mg, 6.84 mmol, 3.0 equiv) and trans-N,N'-dimethylcyclohexane-1,2-diamine (**S42**, 32 mg, 0.2 mmol, 0.09 equiv). The reaction mixture was degassed under N_2 atmosphere for 20 mins, followed by the addition of copper iodide (87 mg, 0.45 mmol, 0.2 equiv). Then reaction mixture was then irradiated in the microwave at 90 °C for 1 h. After cooling to r.t., the reaction mixture was diluted with water (40 mL) and extracted with EtOAc (25 mL × 3). The combined organic extracts were dried over Na_2SO_4 and concentrated under reduced pressure to afford 4-chloro-2-(furan-2-yl)-7-(pyridin-4-yl)-7H-pyrrolo[2,3-d]pyrimidine (**S43**, 300 mg, 44%) as a white solid.

TLC (EtOAc:hexane, 1:1 v/v): Rf = 0.30; ESI MS (m/z): 297.2 [M+H]+; 1H NMR (400 MHz, CDCl3): δ 8.81 (bs, 2H), 7.97 (d, J = 4.4 Hz, 2H), 7.65 (t, J = 2.8 Hz, 1H), 7.39 (d, J = 2.8 Hz, 1H), 6.86 (d, J = 3.2 Hz, 1H), 6.60 (s, 1H), 0.98 – 0.89 (m, 2H), 0.71 (m, 1H); LC/MS: rt = 3.10 min, 99 % peak area at 254 nm (LCMS-09, method B).

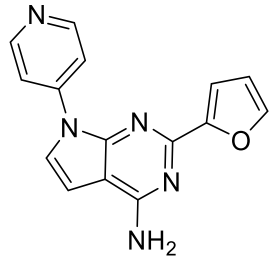

Compound 9

A solution of 4-chloro-2-(furan-2-yl)-7-(pyridin-4-yl)-7H-pyrrolo[2,3-d]pyrimidine (**S43**, 300 mg, 1.01 mmol) in 4% NH_3 in methanol (10.0 mL) was stirred at 50 °C for 16 h. After this time, the reaction mixture was concentrated under reduced pressure. The crude solid was azeotroped with hexanes (3 x 5 mL) to remove access ammonia to give the crude product (320 mg). The crude material was purified by RP-HPLC to give 2-(furan-2-yl)-7-(pyridin-4-yl)-7H-pyrrolo[2,3-d] pyrimidin-4-amine (**Compound 9**, 105.3 mg, 38%) as a white solid.

PREP HPLC Method: The compound was purified on Shimadzu LC-20AP and UV detector. The column used was X-SELECT PHENYL HEXYL (250*19) MM, 5µ. Column flow was 13.0 ml/min. Mobile phase were used (A) 5 mmABC+0.1% NH3 in Water and (B) 100% Acetonitrile. The gradient solvent B was 20-40% over 20 min, then 40-100% over 2 min, 100% over 2 min then 100-20% over 6 min.

TLC (EtOAc): Rf = 0.50; ESI MS (m/z): 278.4 [M+H]+; 1H NMR (400 MHz, DMSO-d6): δ 8.69 (d, J = 4.8 Hz, 2H), 8.24 (d, J = 4.8 Hz, 2H), 7.84 (m, 2H), 7.38 (s, 2H), 7.15 (d, J = 2.4 Hz, 1H), 6.88 (d, J = 3.6 Hz, 1H), 6.64 (t, J = 1.6 Hz, 1H); 13C NMR (101 MHz, DMSO-d6): δ 158.3, 153.6, 152.3, 151.4, 151.3, 145.2,

144.7, 122.8, 115.9, 112.4, 111.7, 103.5, 103.4; LC/MS: rt = 2.25 min, 100v% peak area at 254 nm (LCMS-09, method B).

Supplementary Figures

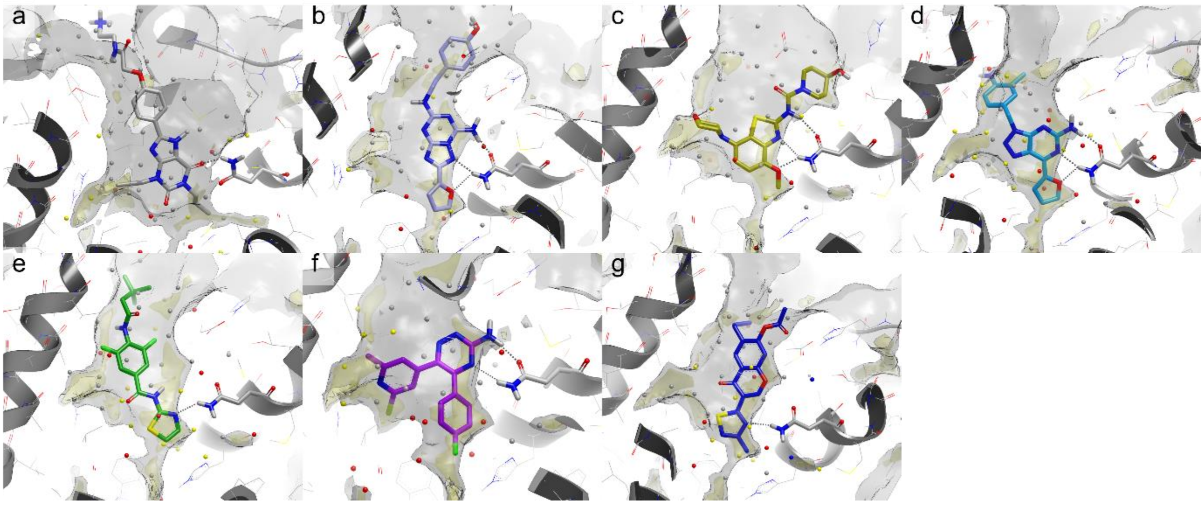

Supplementary Figure 1: A_{2A} receptor crystal structures used for the docking of generated molecules, with PDB codes (a) 3REY, (b) 4EIY, (c) 5OLO, (d) 5OLH, (e) 5OLV, (f) 6GT3 and (g) 6ZDR. The receptor is shown as grey ribbons with residues as thin lines, whereas N253$^{6.55}$ and the co-crystallised ligands are both shown as sticks. GRID maps are shown as transparent solids, with the receptor pocket surface, in terms of how close a ligand carbon atom can go, contoured by a CH3 methyl probe at 1 kcal/mol (in grey) and lipophilic hot spots sub-pockets contoured by a sp2 CH probe (C1 =) probe at -2.8 kcal/mol (in yellow). WaterFLAP water networks calculated on the pseudo-apo binding site (with the ligand removed) are shown as spheres and color-coded by relative energetic scoring in red when predicted free energy (ΔG) is higher than 3.0 kcal/mol, yellow when ΔG is between 1.5 and 3.0 kcal/mol, grey if ΔG is between –2.0 and 1.5 kcal/mol, and blue when $\Delta G < -2.0$ kcal/mol.

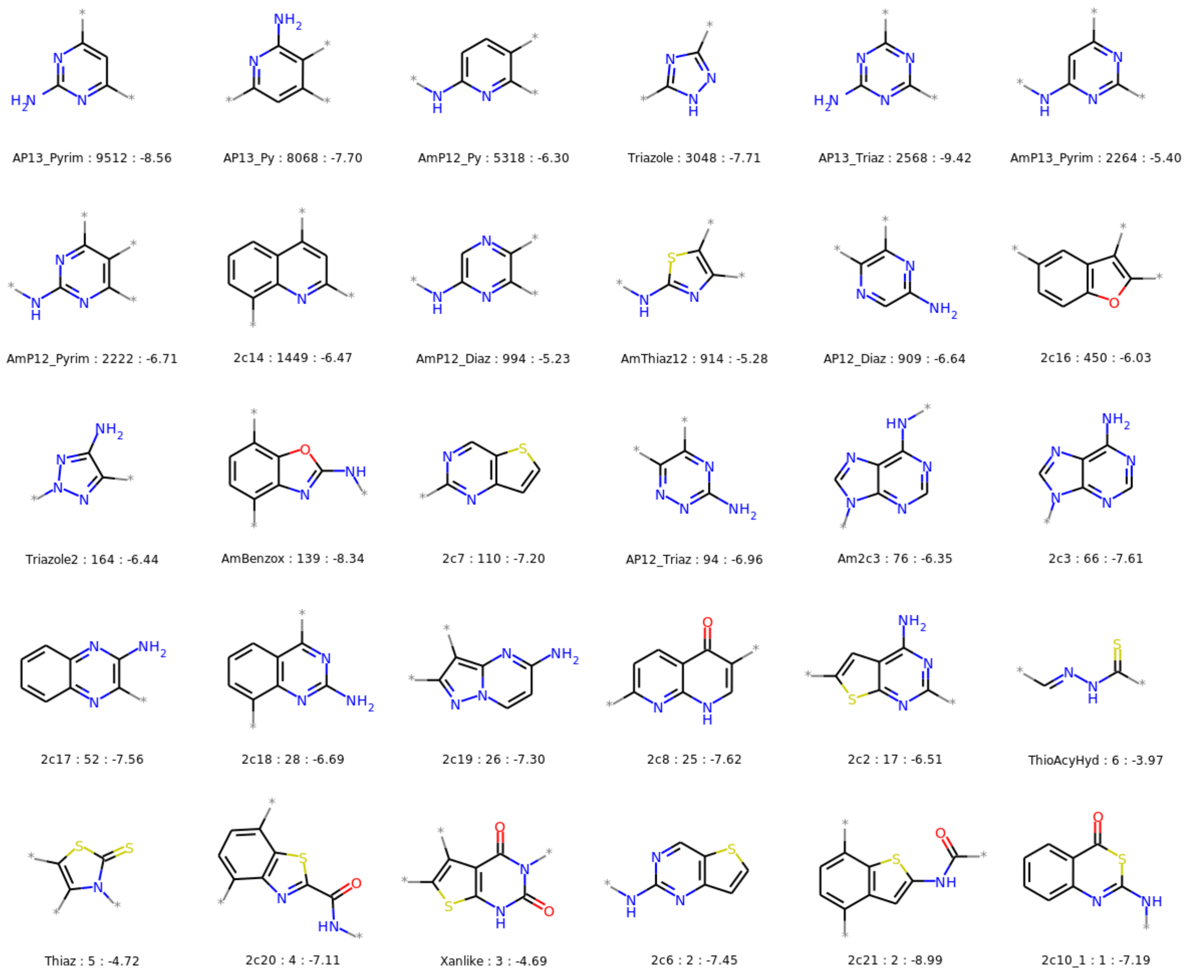

Supplementary Figure 2: Known A_{2A} chemotypes rediscovered. These are in total over all experiments in this work including the name, number rediscovered and the average docking score over all molecules containing the chemotype. Source data are provided as a Source Data file.

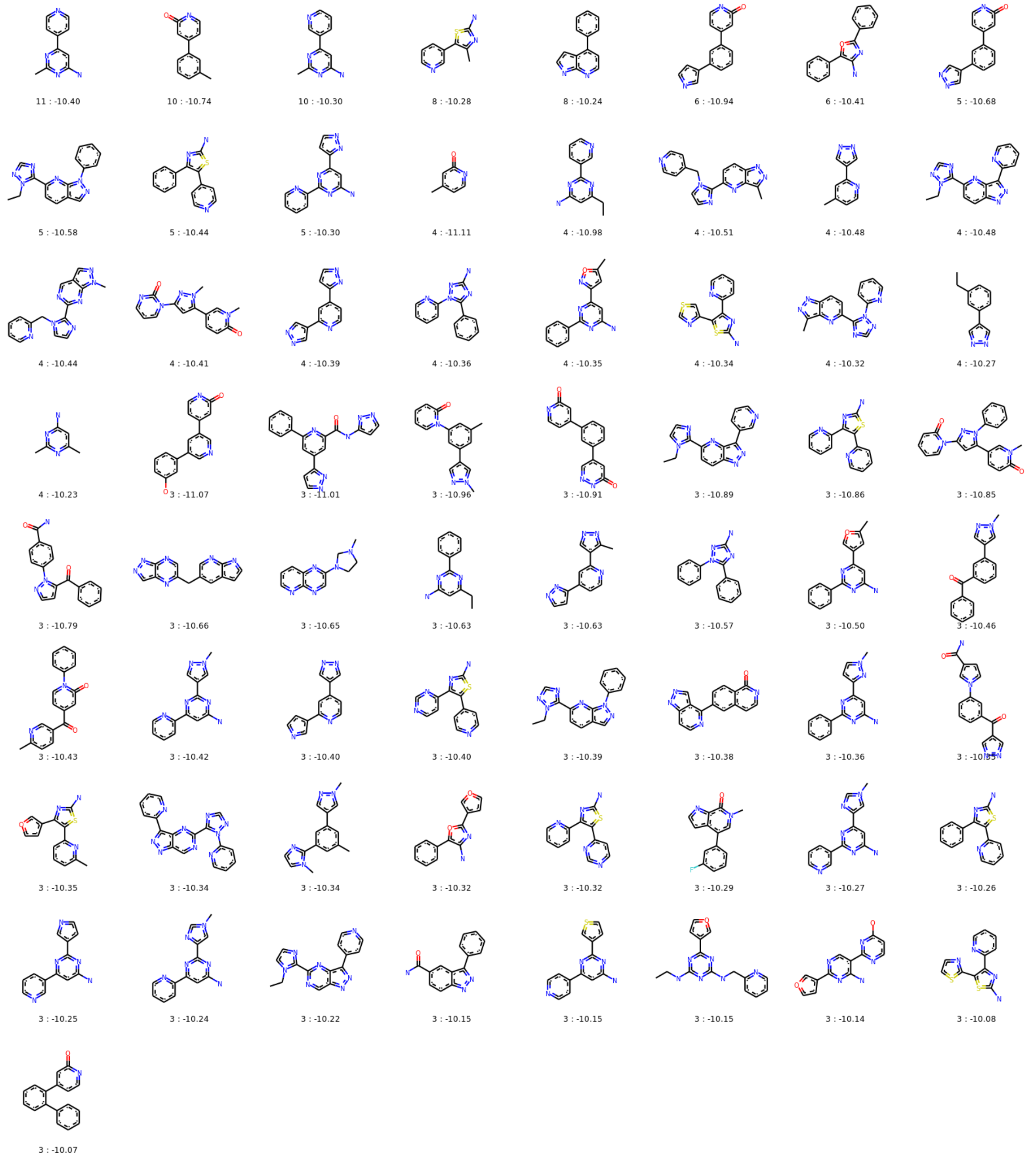

Supplementary Figure 3: Novel chemotypes (represented as Bemis-Murcko scaffolds) with respect to known A_{2A} chemistry. These are in total over all experiments in this work including the number of molecules in each cluster and the average docking score over all molecules in the cluster. Source data are provided as a Source Data file.

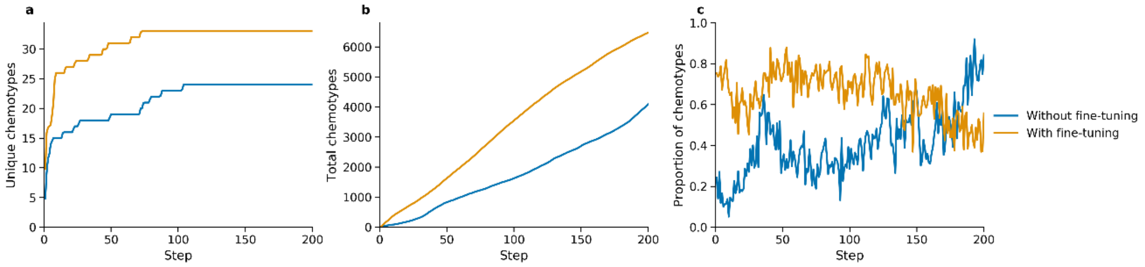

Supplementary Figure 4: The effect of fine-tuning the CLM on A_{2A} active molecules prior to RL on the test system 4EIY. (a) Number of unique A_{2A} chemotypes rediscovered based on whether the CLM was previously fine-tuned or not. (b) Total number of *de novo* molecules containing known A_{2A} chemotypes based on whether the CLM was previously fine-tuned or not. (c) Proportion of molecules containing known A_{2A} ligands per step during optimisation depending on whether the CLM was previously fine-tuned or not. Source data are provided as a Source Data file.

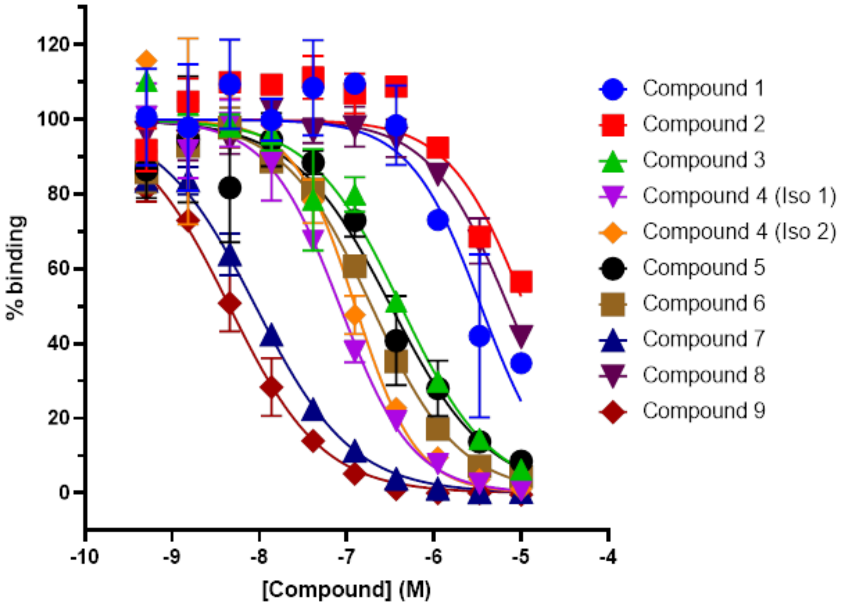

Supplementary Figure 5: Compound affinity estimates using the radioligand binding of [3H]-ZM24385 to human recombinant A_{2A} receptor. Two technical replicates (n=2) were conducted with the mean and standard deviation shown (functional activity is confirmed with (n=3) technical replicates in Figure 7). Source data are provided as a Source Data file.

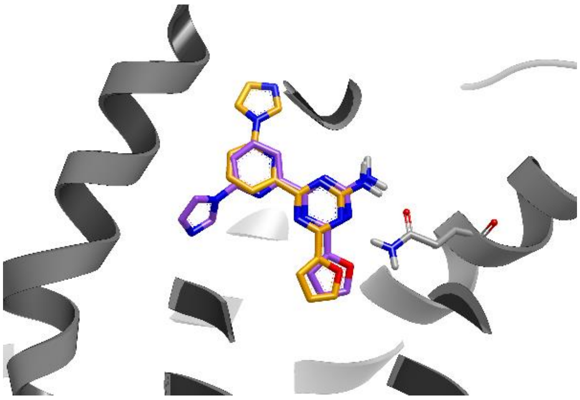

Supplementary Figure 6: A_{2A} receptor crystal structure bound to compound 7 overlayed with the original docking pose of the compound obtained against 6GT3. The receptor is shown as grey ribbons with N253$^{6.55}$, the co-crystallised ligand shown in stick representation with carbons coloured in orange, and docked pose of compound 7 shown in stick representation with purple coloured carbons.

Supplementary Table 1: The compounds that underwent synthesis and experimental validation, and their novelty compared to most similar known A_{2A} ligands, training set compounds, or commercially available vendor compounds. Rediscovered chemotypes are shown with their name below, meanwhile the most similar compounds are shown with their Tanimoto similarity to the respective selected compound. Source data are provided as a Source Data file.

Compound	Structure	Known chemotype	Most similar A_{2A} ligand	Most similar training compound	Most similar commerically available vendor library compound
1	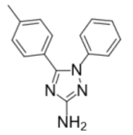		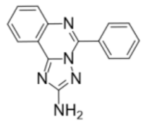 0.43	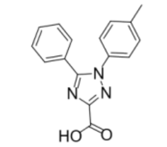 0.55	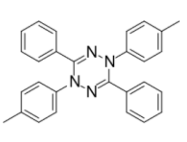 0.635
2	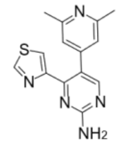	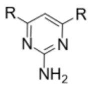 AP13_Pyrim	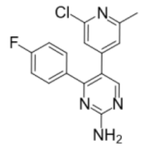 0.47	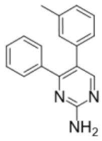 0.42	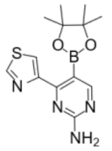 0.48
3	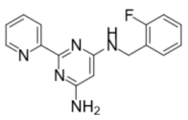	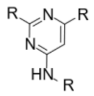 AmP13_Pyrim	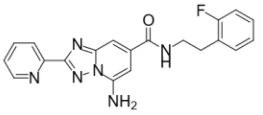 0.43	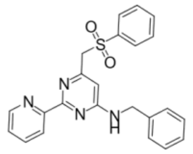 0.45	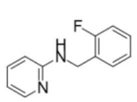 0.63
4	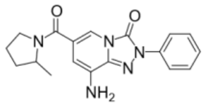		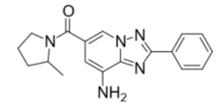 0.61	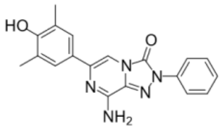 0.45	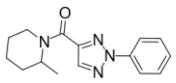 0.57
5	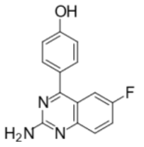	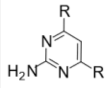 AP13_Pyrim	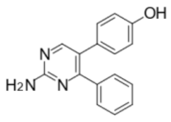 0.44	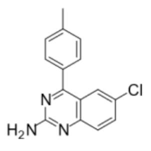 0.54	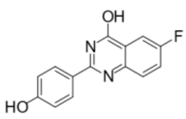 0.53
6	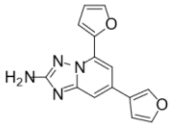		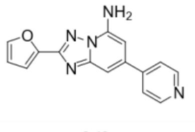 0.49	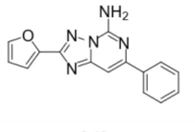 0.43	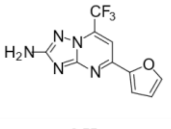 0.55
7	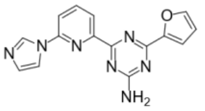	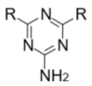 AP13_Triaz	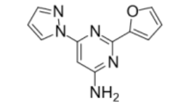 0.41	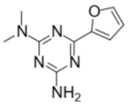 0.42	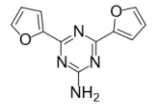 0.50
8	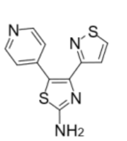		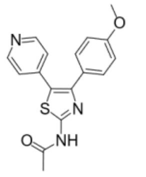 0.34	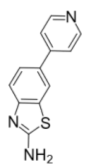 0.38	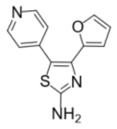 0.64
9	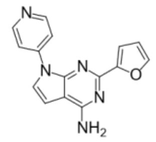		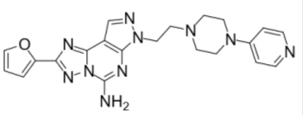 0.43	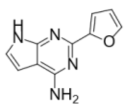 0.42	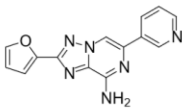 0.46
