# Supplementary material for: Identification of nanomolar adenosine A2A receptor ligands using reinforcement learning and structure-based drug design
Source: Nat Commun. 2025 Jul 1;16:5485. doi: 10.1038/s41467-025-60629-0 (PMC12216625; doi:10.1038/s41467-025-60629-0)
Supplement: Supplementary file 2 — Reporting Summary [file 41467_2025_60629_MOESM2_ESM.pdf]

nature portfolio

Corresponding author(s): Francesca Deflorian
Last updated by author(s): Mar 9, 2025

Reporting Summary

Nature Portfolio wishes to improve the reproducibility of the work that we publish. This form provides structure for consistency and transparency in reporting. For further information on Nature Portfolio policies, see our Editorial Policies and the Editorial Policy Checklist.

Statistics

For all statistical analyses, confirm that the following items are present in the figure legend, table legend, main text, or Methods section.

n/a	Confirmed	
☐	☒	The exact sample size (*n*) for each experimental group/condition, given as a discrete number and unit of measurement
☒	☐	A statement on whether measurements were taken from distinct samples or whether the same sample was measured repeatedly
☒	☐	The statistical test(s) used AND whether they are one- or two-sided<br>*Only common tests should be described solely by name; describe more complex techniques in the Methods section.*
☒	☐	A description of all covariates tested
☒	☐	A description of any assumptions or corrections, such as tests of normality and adjustment for multiple comparisons
☐	☒	A full description of the statistical parameters including central tendency (e.g. means) or other basic estimates (e.g. regression coefficient) AND variation (e.g. standard deviation) or associated estimates of uncertainty (e.g. confidence intervals)
☐	☒	For null hypothesis testing, the test statistic (e.g. *F*, *t*, *r*) with confidence intervals, effect sizes, degrees of freedom and *P* value noted<br>*Give P values as exact values whenever suitable.*
☒	☐	For Bayesian analysis, information on the choice of priors and Markov chain Monte Carlo settings
☒	☐	For hierarchical and complex designs, identification of the appropriate level for tests and full reporting of outcomes
☒	☐	Estimates of effect sizes (e.g. Cohen's *d*, Pearson's *r*), indicating how they were calculated

Our web collection on statistics for biologists contains articles on many of the points above.

Software and code

Policy information about availability of computer code

Data collection	The code used for data generation is available open-source under MIT license. The SMILES-RNN repository [https://github.com/MorganCThomas/SMILES-RNN] contains code to train the generative model and conduct reinforcement learning. The MolScore repository [https://github.com/MorganCThomas/MolScore] was used to define the objective functions that scored the de novo molecules. Both repositories have been been described in previous peer-reviewed publications in the Journal of Cheminformatics. This code was executed using Python (version 3.6.13).
Data analysis	Python (version 3.6.10) and the open-source RDKit software (version 2020.03.3.0) was used for data analysis.

For manuscripts utilizing custom algorithms or software that are central to the research but not yet described in published literature, software must be made available to editors and reviewers. We strongly encourage code deposition in a community repository (e.g. GitHub). See the Nature Portfolio guidelines for submitting code & software for further information.

Data

Policy information about availability of data

All manuscripts must include a data availability statement. This statement should provide the following information, where applicable:

- Accession codes, unique identifiers, or web links for publicly available datasets
- A description of any restrictions on data availability
- For clinical datasets or third party data, please ensure that the statement adheres to our policy

The crystal structure data of Compound 7 and Compound 9 have been deposited in the PDB under the accession codes 9H2X [http://doi.org/10.2210/pdb9H2X/pdb] and 9H37 [http://doi.org/10.2210/pdb9H37/pdb], respectively. The prior training dataset and pre-trained neural network weights are available on GitHub [https://github.com/MorganCThomas/SMILES-RNN] or Zenodo [https://doi.org/10.5281/zenodo.11356193]. Source data are provided with this paper.

Research involving human participants, their data, or biological material

Policy information about studies with human participants or human data. See also policy information about sex, gender (identity/presentation), and sexual orientation and race, ethnicity and racism.

Reporting on sex and gender	Not applicable.
Reporting on race, ethnicity, or other socially relevant groupings	Not applicable.
Population characteristics	Not applicable.
Recruitment	Not applicable.
Ethics oversight	Not applicable.

Note that full information on the approval of the study protocol must also be provided in the manuscript.

Field-specific reporting

Please select the one below that is the best fit for your research. If you are not sure, read the appropriate sections before making your selection.

☒ Life sciences ☐ Behavioural & social sciences ☐ Ecological, evolutionary & environmental sciences

For a reference copy of the document with all sections, see nature.com/documents/nr-reporting-summary-flat.pdf

Life sciences study design

All studies must disclose on these points even when the disclosure is negative.

Sample size	Not applicable.
Data exclusions	No data were excluded.
Replication	Experimental assays were replicated n=3 times.
Randomization	Not applicable.
Blinding	Not applicable.

Reporting for specific materials, systems and methods

We require information from authors about some types of materials, experimental systems and methods used in many studies. Here, indicate whether each material, system or method listed is relevant to your study. If you are not sure if a list item applies to your research, read the appropriate section before selecting a response.

Materials & experimental systems

n/a	Involved in the study
☒	☐ Antibodies
☐	☒ Eukaryotic cell lines
☒	☐ Palaeontology and archaeology
☒	☐ Animals and other organisms
☒	☐ Clinical data
☒	☐ Dual use research of concern
☒	☐ Plants

Methods

n/a	Involved in the study
☒	☐ ChIP-seq
☒	☐ Flow cytometry
☒	☐ MRI-based neuroimaging

Eukaryotic cell lines

Policy information about cell lines and Sex and Gender in Research

Cell line source(s)	CHO-K1, Source ATCC, Cat. no CCL-61. Sex=Female.
Authentication	ATCC comprehensively performs authentication and quality-control tests on all distribution lots of cell lines.
Mycoplasma contamination	CHO-K1 (as annotated as MP00099995 below) was confirmed to be mycoplasma free at Eurofins Genomics.
Commonly misidentified lines (See ICLAC register)	None.

Plants

Seed stocks	Not applicable.
Novel plant genotypes	Not applicable.
Authentication	Not applicable.
